# Intra and post-operative complications observed with femtosecond laser-assisted cataract surgery versus conventional phacoemulsification surgery: a systematic review and meta-analysis

**DOI:** 10.1186/s12886-019-1190-2

**Published:** 2019-08-09

**Authors:** Jinhua Wang, Fanfan Su, Yong Wang, Yao Chen, Qiao Chen, Fen Li

**Affiliations:** 1 Department of Ophthalmology, Jingzhou Central Hospital, Jingzhou, Hubei China; 2 Department of Cataract, Wuhan Aier Eye Hospital, Wuhan, China

**Keywords:** Femtosecond laser-assisted cataract surgery, Conventional phacoemulsification surgery, Post-operative complications

## Abstract

**Background:**

In this analysis, we aimed to systematically compare the complications which were associated with femtosecond laser-assisted cataract surgery (FLACS) versus the conventional phacoemulsification surgery (CPE).

**Methods:**

Commonly used search databases, specifically MEDLINE, Cochrane Central, EMBASE, and http://www.clinicaltrials.gov were carefully searched for English publications comparing FLACS versus CPE. The selected endpoints which were assessed included incomplete capsulotomy, anterior capsulotomy tag, anterior capsule tear, posterior capsule tear, injury to the descemet’s membrane, zonular dialysis, vitreous loss, macular or corneal edema, and elevated intra-ocular pressure. Statistical analysis was carried out by the latest version of the RevMan software (version 5.3) and represented by risk ratios (RR) with 95% confidence intervals (CI).

**Results:**

A total number of 7156 participants were included. Three thousand five hundred and fifty four (3554) participants were assigned to the FLACS group. The risks for incomplete capsulotomy, anterior capsulotomy tag, and anterior capsular tear were significantly higher with FLACS (RR: 22.42, 95% CI: 4.53–110.82; *P* = 0.0001), (RR: 33.07, 95% CI: 6.53–167.56; P = 0.0001) and (RR: 4.74, 95% CI: 2.59–8.68; *P* = 0.00001) respectively. The risks for macular/corneal edema (RR: 2.05, 95% CI: 1.18–3.55; *P* = 0.01) and elevated intra-ocular pressure (RR: 3.24, 95% CI: 1.55–6.78; *P* = 0.002) were also significantly higher with FLACS. However, the risks for impaired descemet’s membrane (RR: 0.95, 95% CI: 0.61–1.47; *P* = 0.80), zonular dialysis (RR: 0.40, 95% CI: 0.06–2.72; *P* = 0.35), vitreous loss (RR: 0.09, 95% CI: 0.01–1.63; *P* = 0.10) and posterior capsular tear (RR: 1.45, 95% CI: 0.23–9.16; *P* = 0.69) were not significantly different.

**Conclusions:**

The current results showed that FLACS did not improve intra/post-operative complications in comparison to CPE. Further larger studies should confirm this hypothesis.

## Background

In this modern developing society, the total number of people undergoing eye surgery is gradually increasing [1]. Agarwal’s et al. recent study based on current and effective features of femtosecond laser-assisted cataract surgery (FLACS) showed that surgeons might now be more confident and patients might be more satisfied with FLACS, but however, a few studies showed that this surgery was not a better option when compared to the manual phacoemulsification in terms of outcomes and complications [2]. Several studies aimed to demonstrate which procedure might come out on top but different opinions were noted [3].

In the large, multi-centered European Registry of Quality Outcomes for Cataract and Refractive Surgery (EUREQUO) study, intraoperative complications for FLACS (0.7%) were similar in comparison to the manual phacoemulsification surgery (0.4%) [4].This same study showed postoperative complications to also have been lower with conventional phacoemulsification (3.4% for FLACS versus 2.3% for phacoemulsification). However, better outcomes were still expected with this new FLACS.

Currently, we aimed to systematically compare the intra/post-operative complications which were associated with FLACS versus the conventional phacoemulsification surgery (CPE).

## Methods

### Search databases

Commonly used search databases, specifically MEDLINE, Cochrane Central, EMBASE, and http://www.clinicaltrials.gov were carefully searched for English publications comparing FLACS versus CPE. Reference lists of several relevant publications were also carefully reviewed.

### Search strategies

The search terms which were used were limited to the following:

- Femtosecond laser-assisted cataract surgery and conventional phacoemulsification surgery;
- Femtosecond laser-assisted cataract surgery and phacoemulsification surgery;
- Femtosecond laser-assisted cataract surgery and complications;
- Femtosecond laser-assisted cataract surgery and post-operative complications;
- Conventional phacoemulsification surgery and complications;
- Conventional phacoemulsification surgery and post-operative complications;
- Conventional phacoemulsification surgery and intra-operative complications;
- Cataract surgeries and post-operative complications;
- Femtosecond laser-assisted cataract and phacoemulsification post-operative complications.

No abbreviation or other short term was used during this search process.

### Inclusion and exclusion criteria

Studies were considered relevant if they compared the complications (intra/peri/post-operative) associated with FLACS versus CPE.

Studies were excluded if:

- They were meta-analyses, systematic reviews, literature reviews and letters of correspondence;
- They did not report peri/intra or post-operative complications associated with FLACS versus CPE;
- They did not report relevant data which could be used in this analysis;
- They were duplicated studies that repeated in several search databases.

### Endpoints which were assessed

The endpoints which were reported in each study have been listed in Table 1.

**Table 1:** Outcomes which were reported

Studies (quality assessment by NOS)	Outcomes reported	Type of complication
Abell2015 (******) [5]	Incomplete capsulotomy, anterior capsulotomy tag, anterior capsule tear, posterior capsule tear, corneal haze, unstable pupil, iris hooks/malyugin ring	Intra-operative
Conrad-Hengerer2013 (******) [6]	Anterior capsule tear, macular edema, elevated intra-ocular pressure	Intra-operative, post-operative
Ewe2015 (******) [7]	Incomplete capsulotomy, anterior capsulotomy tag, anterior capsule tear, posterior capsule tear, corneal haze, corneal epithelial defects, descemet’s membrane trauma, zonular dialysis, mean uncorrected visual acuity, macular edema, ocular hypertension, corneal edema	Peri-operative
Li2018 (*****) [8]	Miosis, descemet’s membrane local detachment, posterior capsular opacification, corneal edema, anterior chamber flare	Intra-operative and post-operative
Mastropasqua2014 (******) [9]	Descemet’s membrane detachment, endothelial gaping, epithelial gaping, endothelial misalignment, epithelial misalignment	Post-operative
Oakley2016 (*****) [10]	Uncorrected visual acuity	Post-operative
Roberts2018 (******) [11]	Anterior capsule tear, descemet membrane tear, iris trauma, residual soft lens matter, posterior capsule tear, vitreous loss, zonular dialysis, corneal edema	Intra-operative
Titiyal2016 (*****) [12]	Vitreous loss, posterior capsular rent, incomplete capsulotomy	Post-operative

Abbreviations: *NOS* Newcastle Ottawa Scale

For quality assessment by the Newcastle Ottawa Scale (NOS), a score is given in terms of stars ranging from a minimum of 1 star (*) to a maximum of 9 stars (*********) based on the quality of the study

The selected endpoints which were assessed included:

- Incomplete capsulotomy;
- Anterior capsulotomy tag;
- Anterior capsule tear;
- Posterior capsule tear;
- Descemet’s membrane impairment;
- Zonular dialysis;
- Vitreous loss;
- Macular or corneal edema;
- Elevated intra-ocular pressure .

### Data extraction and quality assessment

Relevant data were carefully extracted by six independent reviewers. Data included the complications which were assessed with the corresponding number of events, the type of study (randomized or non-randomized prospective), the total number of participants which were assigned to the FLACS and CPE, and the methodological quality of the studies.

The methodological quality of each study was assessed using the Newcastle Ottawa Scale (NOS) [13]. A maximum score of 9 stars was allotted. Scores allotted were based on the quality of the study. The scores have been listed in Table 1.

### Statistical analysis

The statistical analysis in this research paper was carried out by the latest version of the RevMan software (version 5.3). Risk ratios (RR) with 95% confidence intervals (CI) were used to represent the data following statistical analysis.

During this analysis, heterogeneity was assessed first of all by the Q statistic test whereby a subgroup analytical result with a *P* value less or equal to 0.05 was considered as statistically significant and a result with a P value greater than 0.05 was considered statistically insignificant.

In addition, heterogeneity was also assessed by the I^2^ test. In this case, the lower the I^2^ value, the lower the heterogeneity, and in contrast, heterogeneity increased with an increasing I^2^ value.

A fixed statistical effect model was used if the I^2^ value was less than 50% or else, a random statistical effect model was used.

Sensitivity analysis was also carried out following the statistical analysis to observe for any significant change and any particular influence of any specific study on the final results.

In addition, publication bias was assessed through visual observation of the funnel plots.

### Ethical approval

This analysis did not involve research with human or animal participants carried out by any of the authors. Hence, an ethical approval was not required for this study.

## Results

### Search outcomes

A total number of 544 studies were obtained from search databases (PRISMA guideline) [14]. A preliminary assessment was carried out where 486 studies were eliminated on a one-time assessment due to irrelevance.

Fifty-eight (58) full-text articles were carefully assessed for eligibility.

Further eliminations were carried out based on the following reasons:

- Meta-analyses (1);
- Studies that did not report intra or post-operative complications (6);
- Studies involving data that were not suitable for this research (8);
- Studies whereby a control group was absent (5);
- Duplicated studies since they repeated themselves in several different search databases (30).

**Fig. 1:**
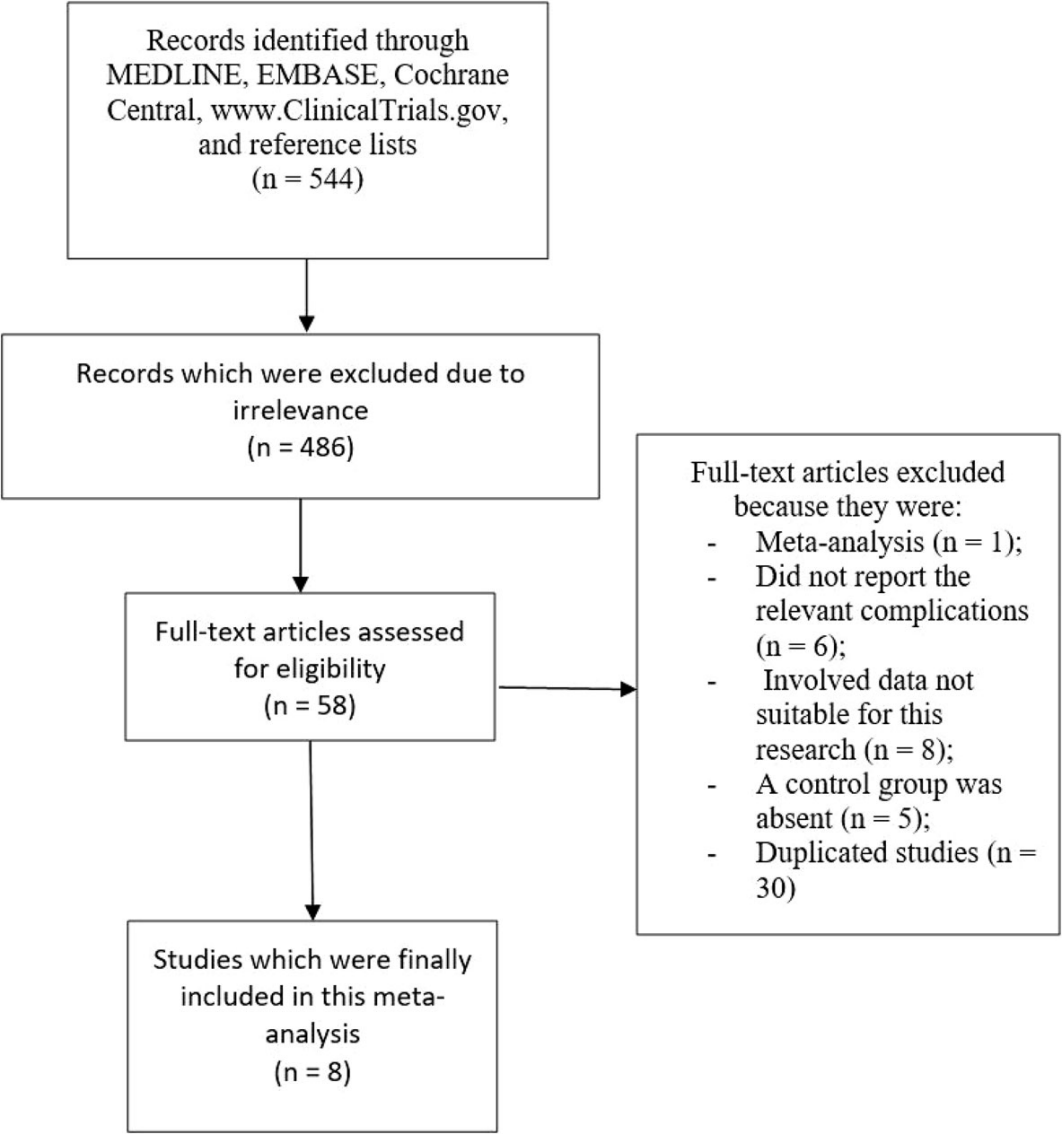
Flow diagram showing the study selection

Finally, 8 prospective studies (randomized and non-randomized) [5–12] were selected to be included in this analysis as shown in Fig. 1.

### General and baseline properties of the studies

The general properties of the studies have been listed in Table 2.

**Table 2:** General properties of the studies

Studies	Total no of participants undergoing FLACS (n)	Total no of participants undergoing CPE (n)	Time period of patients’ enrollment (years)	Type of study
Abell2015 [5]	1852	2228	2012–2013	NR prospective
Conrad-Hengerer2013 [6]	73	73	-	Randomized prospective
Ewe2015 [7]	988	888	2012–2014	NR prospective
Li2018 [8]	48	48	2016–2017	Randomized prospective
Mastropasqua2014 [9]	30	30	-	Randomized prospective
Oakley2016 [10]	323	95	2012–2014	NR prospective
Roberts2018 [11]	200	200	-	Randomized prospective
Titiyal2016 [12]	40	40	-	NR prospective
Total no (n)	3554	3602	-	

Abbreviations: *FLACS* femtosecond laser-assisted cataract surgery, *CPE* conventional phacoemulsification surgery, *NR* non-randomized

A total number of 7156 participants were included in this analysis. Three thousand five hundred and fifty four (3554) participants were assigned to the FLACS group whereas 3602 participants were assigned to the CPE group as shown in Table 2. The participants were enrolled from the years 2012 to 2017. The studies were either randomized or non-randomized prospective studies.

### Main results

**Fig. 2:**
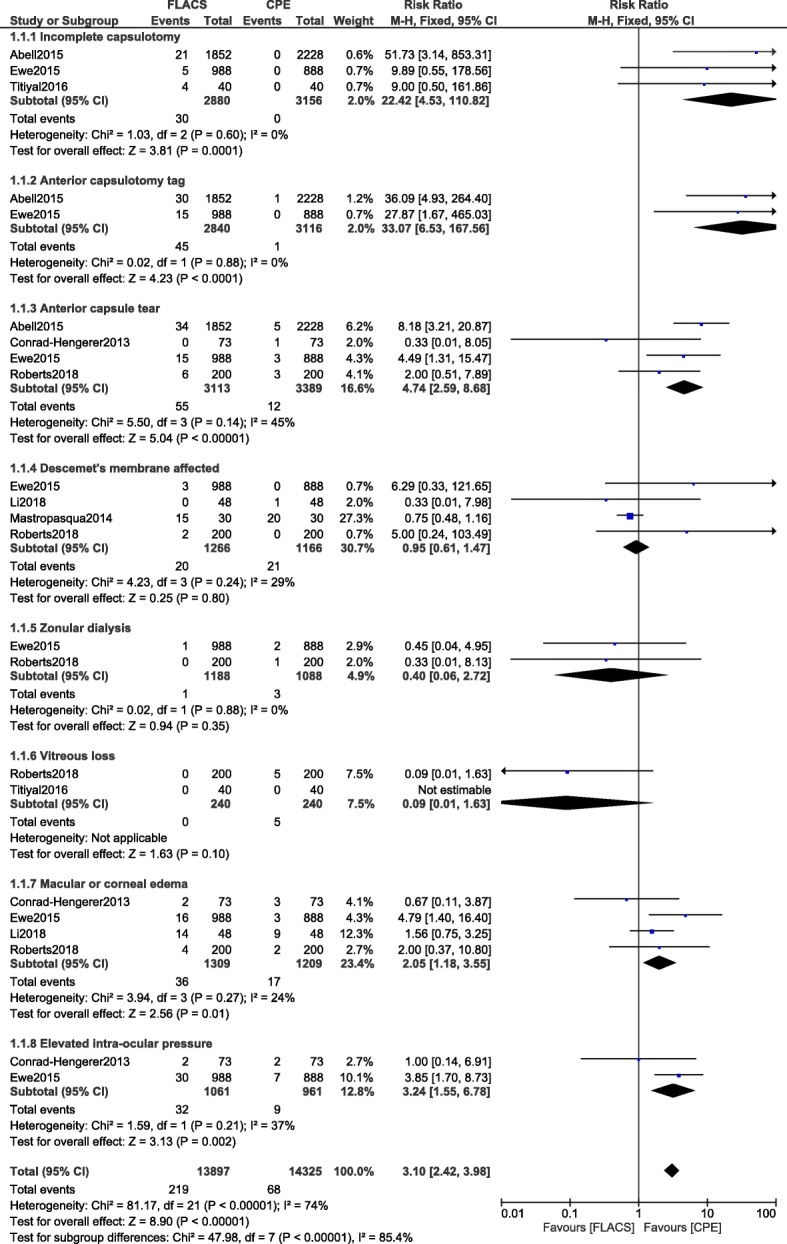
Intra/post-operative complications associated with femtosecond laser assisted cataract surgery versus conventional phacoemulsification surgery (part I)

Following the statistical analysis, the risks for incomplete capsulotomy, anterior capsulotomy tag, and anterior capsular tear were significantly higher with FLACS (RR: 22.42, 95% CI: 4.53–110.82; *P* = 0.0001), (RR: 33.07, 95% CI: 6.53–167.56; P = 0.0001) and (RR: 4.74, 95% CI: 2.59–8.68; *P* = 0.00001) respectively as shown in Fig. 2. The risks for macular/corneal edema (RR: 2.05, 95% CI: 1.18–3.55; *P* = 0.01) and elevated intra-ocular pressure (RR: 3.24, 95% CI: 1.55–6.78; *P* = 0.002) were also significantly higher with FLACS.

However, impaired descemet’s membrane (RR: 0.95, 95% CI: 0.61–1.47; *P* = 0.80), zonular dialysis (RR: 0.40, 95% CI: 0.06–2.72; *P* = 0.35) and vitreous loss (RR: 0.09, 95% CI: 0.01–1.63; *P* = 0.10) were not significantly different as shown in Fig. 2.

**Fig. 3:**
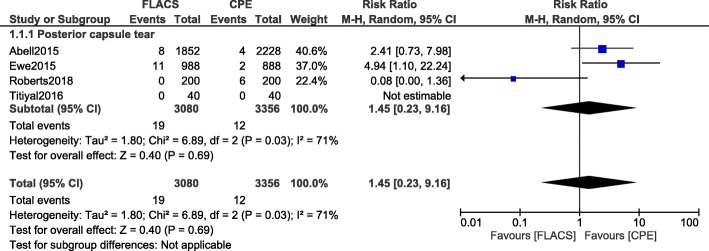
Intra/post-operative complications associated with femtosecond laser assisted cataract surgery versus conventional phacoemulsification surgery (part II)

The risk for posterior capsular tear (RR: 1.45, 95% CI: 0.23–9.16; *P* = 0.69) was also similar as demonstrated in Fig. 3.

Sensitivity analysis was also carried out. Consistent results were obtained throughout with the exception of subgroup assessing for ‘posterior capsular tear’. When study Roberts2018 was excluded and another analysis was carried out, the risk for posterior capsular tear (RR: 3.34, 95% CI: 1.32–8.47; *P* = 0.01) was significantly higher with FLACS. In addition, when study Ewe2015 was excluded, the risk for macular/corneal edema (RR: 1.43, 95% CI: 0.76–2.67; *P* = 0.26) was not significantly different.

The results have been listed in Table 3.

**Table 3:** Results of this analysis

Complications which were analyzed	RR with 95% CI	*P* value	I^2^ value (%)
Incomplete capsulotomy	22.42 [4.53–110.82]	0.0001	0
Anterior capsulotomy tag	33.07 [6.53–167.56]	0.0001	0
Anterior capsule tear	4.74 [2.59–8.68]	0.00001	45
Descemet’s membrane affected	0.95 [0.61–1.47]	0.80	29
Zonular dialysis	0.40 [0.06–2.72]	0.35	0
Vitreous loss	0.09 [0.01–1.63]	0.10	0
Macular or corneal edema	2.05 [1.18–3.55]	0.01	24
Elevated intra-ocular pressure	3.24 [1.55–6.78]	0.002	37
Posterior capsule tear	1.45 [0.23–9.16]	0.69	71

Abbreviations: *RR* risk ratios, *CI* confidence intervals

**Fig. 4:**
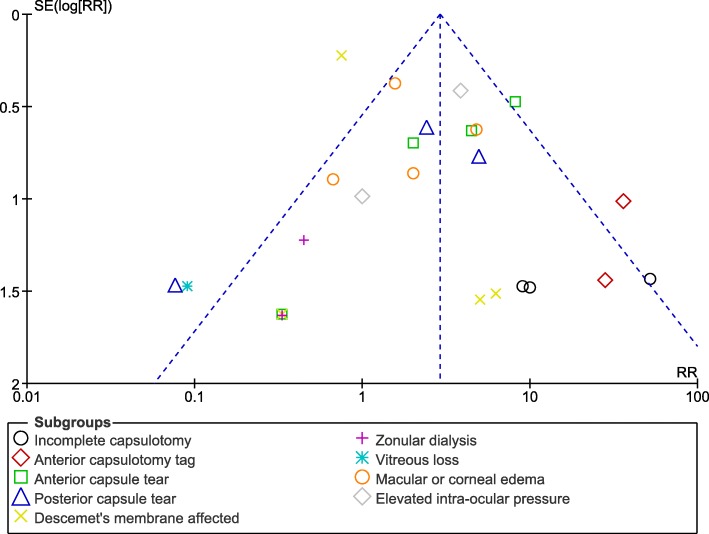
Funnel plot representing publication bias

Low evidence of publication bias was observed as demonstrated by the funnel plot in Fig. 4.

## Discussion

In this current analysis, we compared the intra and post-operative complications which were associated with FLACS versus CPE. The results showed no improvement in complications with the former. FLACS was associated with significantly higher risks of incomplete capsulotomy, anterior capsulotomy tag and anterior capsular tear. The risks for macular/corneal edema, and elevated intra-ocular pressure were also significantly higher with FLACS.

As previously mentioned in the introduction section, in the EUREQUO study [2], intraoperative complications for FLACS (0.7%) were similar in comparison to the manual phacoemulsification surgery (0.4%). The same study showed postoperative complications to also be lower with CPE (3.4% for FLACS versus 2.3% for phacoemulsification) further supporting the results of this analysis.

In addition, another meta-analysis also showed posterior capsular tear to be significantly higher with FLACS in comparison to the manual phacoemulsification surgery again supporting the results of this current analysis [15].

Even if this current analysis did not assess for astigmatism, a retrospective study showed astigmatic changes to be more common with FLACS [16].

However, we should not forget the fact that complications such as vitreous loss might be reduced with FLACS [17] and therefore, its complications should not be overestimated but instead, we should also pay attention to its beneficial features. Also, FLACS using the LenSx laser system might achieve better results in a real world setting [18].

Even though the total number of participants undergoing cataract surgery was sufficient to reach a conclusion, the number of participants were distributed during the subgroup analyses, and hence, only less number of patients participated in each subgroup analysis. However, we could not improve this limitation since only a few original research articles were published on this particular topic. Because of this same reason, data from different randomized and non-randomized prospective studies were pooled together during analysis. There was no other choice or a very biased result with lack of strength would have been obtained. Also, due to a shortage of studies, we included one study comparing cystotome-assisted prechop phacoemulsification surgery versus CPE in this analysis. This might not affected the results to a large extent since the number of participants in the study was very less. Also, there was no specific follow-up time period post-operatively. All the studies which were included in this analysis did not involve the same follow-up time period.

## Conclusions

This current results showed that FLACS did not improve intra/post-operative complications in comparison to CPE. Further larger studies should confirm this hypothesis.

## Data Availability

All data and materials used in this research are freely available. References have been provided.

## Abbreviations

CI: Confidence intervals
CPE: Conventional phacoemulsification surgery
FLACS: Femtosecond laser-assisted cataract surgery
RR: Risk ratios

## References

[1] Cox JT, Subburaman G, Munoz B, Friedman DS, Ravindran RD. Visual acuity outcomes after cataract surgery: high- vs. low-volume surgeons. Ophthalmology. 2019; Apr 8. pii: S0161-6420(18)31302-2. 10.1016/j.ophtha.2019.03.033 30974182

[2] Agarwal A, Jacob S (2017). Current and effective advantages of femto phacoemulsification. Curr Opin Ophthalmol.

[3] Ewe SY, Abell RG, Vote BJ (2018). Femtosecond laser-assisted versus phacoemulsification for cataract extraction and intraocularlens implantation: clinical outcomes review. Curr Opin Ophthalmol.

[4] Manning S, Barry P, Henry Y, Rosen P, Stenevi U, Young D, Lundström M (2016). Femtosecond laser-assisted cataract surgery versus standard phacoemulsification cataractsurgery: study from the European registry of quality outcomes for cataract and refractive surgery. J Cataract Refract Surg.

[5] Abell RG, Darian-Smith E, Kan JB, Allen PL, Ewe SY, Vote BJ (2015). Femtosecond laser-assisted cataract surgery versus standard phacoemulsification cataractsurgery: outcomes and safety in more than 4000 cases at a single center. J Cataract Refract Surg.

[6] Conrad-Hengerer I, Al Juburi M, Schultz T, Hengerer FH, Dick HB (2013). Corneal endothelial cell loss and corneal thickness in conventional compared with femtosecondlaser-assisted cataract surgery: three-month follow-up. J Cataract Refract Surg.

[7] Ewe SY, Abell RG, Oakley CL, Lim CH, Allen PL, McPherson ZE, Rao A, Davies PE, Vote BJ (2016). A comparative cohort study of visual outcomes in femtosecond laser-assisted versus phacoemulsification cataract surgery. Ophthalmology..

[8] Li X, He Y, Su T, Tian Y, Wang Y, Xia X, Song W (2018). Comparison of clinical outcomes between cystotome-assisted prechop phacoemulsificationsurgery and conventional phacoemulsification surgery for hard nucleus cataracts: a CONSORT-compliant article. Medicine (Baltimore).

[9] Mastropasqua L, Toto L, Mastropasqua A, Vecchiarino L, Mastropasqua R, Pedrotti E, Di Nicola M (2014). Femtosecond laser versus manual clear corneal incision in cataract surgery. J Refract Surg.

[10] Oakley CL, Ewe SY, Allen PL, Vote BJ (2016). Visual outcomes with femtosecond laser-assisted cataract surgery versus conventional cataractsurgery in toric IOL insertion. Clin Exp Ophthalmol.

[11] Roberts HW, Wagh VK, Sullivan DL, Hidzheva P, Detesan DI, Heemraz BS, Sparrow JM, O'Brart DPS (2019). A randomized controlled trial comparing femtosecond laser-assisted cataract surgery versus conventional phacoemulsification surgery. J Cataract Refract Surg.

[12] Titiyal JS, Kaur M, Singh A, Arora T, Sharma N (2016). Comparative evaluation of femtosecond laser-assisted cataract surgery and conventionalphacoemulsification in white cataract. Clin Ophthalmol.

[13] Wells GA, Shea B, O'Connell D, Peterson J, Welch V, Losos M (2009). TheNewcastle-Ottawa scale (NOS) for assessing the quality if nonrandomized studies in meta-analyses.

[14] Liberati A, Altman DG, Tetzlaff J, Mulrow C, Gøtzsche PC, Ioannidis JP, Clarke M, Devereaux PJ, Kleijnen J, Moher D (2009). The PRISMA statement for reporting systematic reviews and meta-analyses of studies that evaluate healthcareinterventions: explanation and elaboration. BMJ.

[15] Popovic M, Campos-Möller X, Schlenker MB, Ahmed II (2016). Efficacy and safety of femtosecond laser-assisted cataract surgery compared with ManualCataract surgery: a meta-analysis of 14 567 eyes. Ophthalmology..

[16] Lee JA, Song WK, Kim JY, Kim MJ, Tchah H (2019). Femtosecond laser-assisted cataract surgery versus conventional phacoemulsification: refractive and aberrometric outcomes with a diffractive multifocal intraocular lens. J Cataract Refract Surg.

[17] Scott WJ, Tauber S, Gessler JA, Ohly JG, Owsiak RR, Eck CD (2016). Comparison of vitreous loss rates between manual phacoemulsification and femtosecond laser-assisted cataract surgery. J Cataract Refract Surg.

[18] Zhang X, Yu Y, Zhang G, Zhou Y, Zhao G, Chen M, Wang Y, Zhu S, Zhang H, Yao K (2019). Performance of femtosecond laser-assisted cataract surgery in Chinese patients with cataract: a prospective, multicenter, registry study. BMC Ophthalmol.

